# Sleep Deprivation and Neuronal Hyperexcitation Share Transcriptomic Signatures

**DOI:** 10.1002/npr2.70150

**Published:** 2026-06-30

**Authors:** Markos Michail Chatzigiannis, Hideo Hagihara, Tsuyoshi Miyakawa

**Affiliations:** ^1^ Department of Systems Medical Science Fujita Health University Graduate School of Medicine Toyoake Aichi Japan; ^2^ Division of Systems Medical Science, Center for Medical Science Fujita Health University Toyoake Aichi Japan

**Keywords:** hyperexcitation, immediate‐early genes, inflammation, sleep deprivation, transcriptome

## Abstract

Although sleep deprivation (SD) is clinically associated with numerous neuropsychiatric disorders, its underlying molecular correlates remain unclear. Because extended wakefulness is accompanied by increased neuronal activity and network firing, SD may be associated with a hyperactive neural state. This study aimed to test the hypothesis that SD shares transcriptomic signatures induced by neuronal hyperexcitation and to identify the gene pathways and cell types associated with these signatures. Publicly available transcriptomic datasets were analyzed, including 32 SD and 23 neuronal hyperexcitation transcriptomic datasets. These datasets were systematically compared using the Running Fisher algorithm across multiple mouse brain regions and rodent neuronal hyperexcitation models. The analysis revealed significant positive transcriptomic overlaps between SD and neuronal hyperexcitation models (*p* ≤ 0.05 in 73% of cross‐model comparisons). In addition, neuronal hyperexcitation datasets collected within 1–12 h after seizure induction showed stronger transcriptomic similarity to SD than those collected 24 h or later. The shared transcriptomic signature was significantly enriched for pathways associated with neuronal plasticity, immune response, and inflammation. Key overexpressed genes common to both conditions included immediate early genes (IEGs) such as Egr1, Fos, and Arc, as well as inflammation‐associated genes such as Ptgs2 and Junb. Comparisons between SD single‐cell and neuronal hyperexcitation datasets indicated that the shared signature was most strongly enriched in microglia and neurons, with additional contributions from endothelial cells and astrocytes. Microglia showed enrichment of stress‐ and immune‐response genes, neurons exhibited IEG and plasticity‐related signatures, and endothelial cells expressed metabolism‐associated genes. Together, these findings indicate that SD is associated with a transcriptomic state resembling acute neuronal hyperexcitation, characterized by activation of neuronal plasticity‐, neuroinflammatory‐, and metabolism‐related pathways. This shared molecular signature provides a transcriptomic framework linking sleep loss to molecular processes implicated in neuropsychiatric disorders and suggests that acute neuronal hyperexcitation‐related molecular processes may contribute to SD‐associated brain dysfunction.

## Abstract 2

This study demonstrated that sleep deprivation induces transcriptomic changes in mice and rats similar to those observed in various models of neuronal hyperexcitation. These findings suggest a potential link between sleep deprivation and neuropsychiatric disorders.

## Graphical abstract

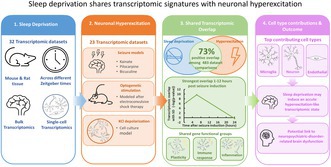

## 1 Introduction

Insufficient sleep affects up to a third of the adult population and is a significant risk factor for various neuropsychiatric disorders, including major depressive disorder, bipolar disorders, Alzheimer's disease, and Parkinson's disease [1, 2, 3, 4, 5]. Notably, epidemiological studies indicate that 40%–83% of patients with psychiatric disorders experience clinically significant sleep disturbances [6, 7, 8]. Another study reported that individuals receiving, on average, 6 or fewer hours of sleep were 2.5 times more likely to report mental distress than individuals who averaged more than 6 h of sleep per night [9]. Furthermore, insufficient sleep is associated with impaired memory, and prolonged sleep deprivation (SD) has been reported to induce psychosis‐like symptoms [10, 11]. Despite extensive behavioral, electrophysiological, and clinical research, the molecular mechanisms by which SD alters brain function remain incompletely understood [12, 13, 14].

Experimental studies have associated SD with neuronal hyperexcitation, a condition characterized by an increased likelihood of neurons being activated by a certain stimulus [15, 16]. Increased brain excitability after SD has been confirmed with electroencephalography (EEG) and transcranial magnetic stimulation (TMS) in both human subjects and animal models [17, 18, 19]. For example, a study using EEG to measure the cortical response to TMS observed a progressively increased response with time awake [19]. Based on such studies, neuronal hyperexcitation has been proposed as a neurophysiological consequence of SD.

Given that neuronal hyperexcitation is associated with SD, it is notable that this state is also a common feature of multiple neuropsychiatric disorders. Neuronal hyperexcitation is a phenomenon associated with epileptic conditions but has also been observed in various other neuropsychiatric disorders such as schizophrenia, intellectual disability, autism spectrum disorders, and Alzheimer's disease [20, 21, 22]. Indeed, evidence for neuronal hyperexcitation includes the higher prevalence of epilepsy in these populations [23, 24], as well as findings from EEG studies [25] and functional magnetic resonance imaging studies [26]. This pathology may be rooted in an excitation/inhibition (E/I) imbalance [20, 27, 28, 29]. Kindling models demonstrate that even small imbalances in excitation, when sustained, can significantly impact brain circuits [30, 31]. In combination, these findings suggest that neuronal hyperexcitation may represent a shared pathophysiological substrate across diverse brain disorders.

Sleep has been proposed to play a critical role in regulating neuronal excitability and synaptic strength. Wakefulness is associated with an overall increase in net neuronal excitability, whereas sleep is thought to promote the renormalization of neuronal excitability [32, 33]. From this perspective, SD may result in the prolonged activation of transcriptomic pathways that are normally regulated by sleep [34, 35, 36]. Because prolonged wakefulness is associated with increased neuronal firing and reduced inhibitory control, sleep loss could engage molecular programs similar to those activated during hyperexcitable brain states. However, despite extensive behavioral and electrophysiological studies, it remains unclear whether SD and neuronal hyperexcitation share common transcriptomic signatures.

Importantly, transcriptomic responses to neuronal hyperexcitation are neither uniform nor static [37, 38, 39]. Instead, they depend on experimental context, particularly the timing relative to excitatory events and the specific cell types involved [40, 41]. Acute phases of hyperexcitation are characterized by rapid induction of immediate early genes (IEGs) and plasticity‐related pathways, whereas later phases are associated with distinct transcriptional profiles, including inflammatory and stress‐related responses [40, 41]. Moreover, neurons, glial cells, and endothelial cells exhibit markedly different transcriptional responses to increased neuronal activity [42]. These observations raise the possibility that SD may resemble specific temporal phases and cellular components of neuronal hyperexcitation rather than representing a generalized hyperexcitable brain state.

A major challenge in testing this hypothesis lies in the substantial heterogeneity among publicly available transcriptomic datasets, including differences in experimental design, brain regions, species, and sequencing platforms. To enable robust comparison across such diverse datasets, we employed the Running Fisher algorithm implemented in the BaseSpace Correlation Engine [43]. This rank‐based, nonparametric method evaluates enrichment of concordantly regulated genes across entire ranked gene lists, rather than relying on arbitrary significance cutoffs, and incorporates both the direction and relative magnitude of expression changes [43]. As such, it is particularly well suited for cross‐platform and cross‐context comparison of transcriptomic signatures, enabling integration of bulk RNA‐sequencing (RNA‐seq), microarray, and single‐cell RNA‐seq (scRNA‐seq) datasets from diverse experimental models [44, 45, 46, 47].

In the present study, we applied this approach to perform a large‐scale comparative transcriptomic analysis of publicly available SD and neuronal hyperexcitation datasets. By systematically screening BaseSpace [43] and the NCBI Gene Expression Omnibus databases, we identified 21 RNA‐seq/microarray and 11 scRNA‐seq SD datasets spanning hippocampus, hypothalamus, cerebellum, and cortex, which were analyzed alongside 23 bulk transcriptomic datasets related to neuronal hyperexcitation. While the SD datasets varied in duration of SD (from 3 to 12 h) and initiation time (zeitgeber time 0–18) and the neuronal hyperexcitation datasets varied in seizure/stimulation models, this heterogeneity was utilized for the detection of molecular signals that are persistent across experimental conditions. To facilitate the comparison of these highly heterogeneous datasets we employed the Running Fisher method, which is a rank‐based and nonparametric approach that allows for the integration of heterogeneous data from different platforms such as bulk RNA‐seq and scRNA‐seq [43]. Using this method, we aimed to determine (1) whether SD shares transcriptomic signatures with neuronal hyperexcitation, (2) whether the degree of similarity depends on timing relative to excitatory events or stimulation paradigms, and (3) which brain cell types contribute most strongly to the observed overlap. Finally, pathway enrichment and cell‐type‐specific analyses of shared gene signatures were used to gain insight into the biological processes and cellular populations underlying the molecular relationship between SD and neuronal hyperexcitation.

## 2 Methods

All the datasets utilized in this study were publicly available gene expression data obtained from the database BaseSpace (Illumina, San Diego, CA) and the Gene Expression Omnibus. A summary of the datasets, including the study name, number of samples, age, and data source, is presented in Table S1. The specific datasets used in each panel of the figures are detailed in Table S2.

### 2.1 Hyperexcitation‐Associated Genes

The following datasets from neuronal hyperexcitation models were used to assess their transcriptomic similarity to SD.

### 2.2 Optogenetic‐Induced Hyperexcitation Datasets

Optogenetically stimulated 3–4‐month‐old mice were used to obtain hyperexcitation‐associated genes [48]. The mice received brief stimulation either 3 or 10 times and were dissected 24 h or 2 weeks after the final stimulation. Gene expression patterns in the hippocampal dentate gyrus were then compared with those of control mice with no stimulation, yielding datasets of differentially expressed genes (DEGs) associated with neuronal hyperexcitation. Repeated stimulation of the dentate gyrus has been shown to induce neuronal hyperexcitation, making this an appropriate model for the present analysis [49, 50].

### 2.3 KCl‐Depolarization Neural Culture Datasets

Data from a study using cortical neuronal cultures from newborn mice (postnatal day 0 (P0), 8 days in vitro (DIV)) and hippocampal neuronal cultures from mouse embryos (embryonic day 16 (E16), 5 DIV) treated with KCl were used as a model of ionic depolarization‐induced hyperexcitation [51]. Gene expression was measured 1 and 6 h after treatment for the cortical neurons, whereas for the hippocampal neurons, measurements were taken 1, 3, and 6 h after treatment. Gene expression in the treated neurons was compared with untreated ones to obtain DEG datasets.

### 2.4 Bicuculline‐Induced Hyperexcitation Datasets

Data from a study using cortical neuronal cultures from embryonic mice (E15, 12 DIV) treated with bicuculline were used as a model of chemically induced hyperexcitation [52]. Six hours after exposure to bicuculline, gene expression in the treated neurons was compared with untreated controls to obtain DEG datasets.

### 2.5 Kainate‐Induced Hyperexcitation Datasets

Data from two studies using rats or mice treated with kainate were used as models of chemically induced hyperexcitation. The first study utilized juvenile rats (P15) [53]. Their hippocampi were dissected 1, 6, and 24 h after seizure induction (SI), and DEG datasets were obtained by comparison with age‐matched controls. The second study used 3‐month‐old adult mice. Hippocampi were dissected at 1, 4, 8, and 24 h after SI, and DEG datasets were generated using age‐matched controls [54].

### 2.6 Pilocarpine‐Induced Hyperexcitation Datasets

Data from two studies using pilocarpine‐treated mice were used as models of chemically induced hyperexcitation. The first study used 6–7‐week‐old mice dissected at 1, 8, or 36 h after SI, and hippocampal DEG datasets were generated by comparison with age‐matched controls [40]. The second study used 7–10‐week‐old mice whose dentate gyri were dissected at 1, 6, or 12 h post‐SI, and DEG datasets were similarly produced using age‐matched controls [55].

### 2.7 Sleep Deprivation Datasets

Four studies were used to represent SD. The first study utilized three inbred mouse strains (AKR/J, C57BL/6J, and DBA/2J), all approximately 2 months old. Mice were sleep‐deprived starting at four different circadian time points: light onset (zeitgeber time (ZT) 0), mid‐light (ZT6), the beginning of the dark (ZT12), and mid‐dark (ZT18) [56]. SD lasted for a total of 6 h. Following SD, the mice were sacrificed, and DEGs in the hippocampi were identified by comparing gene expression profiles with those of non‐sleep‐deprived controls. The second study used 10‐week‐old mice that were sleep‐deprived at ZT0 for 3, 6, 9, or 12 h. Following SD, the hypothalamus and cerebral cortex were dissected, and DEG datasets were generated using procedures similar to the first study [57]. The third study used 2–4‐month‐old mice that were sleep‐deprived at ZT5 for 5 h. Hippocampal DEG datasets were similarly identified by comparison with non‐sleep‐deprived controls [58]. For the cell‐type analysis, single‐cell data from a transcriptomic study of 9–11‐week‐old mice sleep‐deprived for 12 h at ZT0 were used [59]. Differential expression profiles of oligodendrocytes, neurons, microglia, ependymocytes, macrophages, astrocytes, and endothelial cells from the hypothalamus and/or cortex were compared with the neuronal hyperexcitation datasets (Tables S1 and S3).

### 2.8 Developmental Datasets

Transcriptomic data from mice of varying ages were used to investigate the presence of transcriptomic immaturity in the brains of SD mice. In total, developmental data were obtained from three different studies. Two studies provided dentate gyrus transcriptomic data from P8 mice and 33‐week‐old adult mice, which were compared with each other to produce a P8 vs. Adult (33‐week‐old) mice developmental DEG dataset [60, 61]. The third study provided hippocampal data from P30, P15, P7, and P1 mice [62]. Datasets were created by comparing data of each age group within the same study. Thus, datasets such as “P15 vs P30 mice” refer to DEGs in P15 as compared with P30 mice.

### 2.9 Calculation of DEGs

Apart from the pilocarpine, developmental and single‐cell SD datasets, the DEG datasets used in this study are already available in BaseSpace. These datasets are curated by BaseSpace curators using publicly available transcriptomic data (e.g., from NCBI GEO) and are generated by comparing experimental groups with control groups using the criteria of an absolute fold change > 1.2 and a raw *p*‐value < 0.05. For datasets not included in BaseSpace, we generated DEG datasets using the same criteria and manually registered them in BaseSpace. Regardless of how they were registered, all datasets within BaseSpace can be systematically compared and analyzed.

### 2.10 Evaluating the Similarity of Gene Expression Patterns

To determine whether the gene expression changes induced by SD resemble those observed under neuronal hyperexcitation, the DEGs associated with each condition were compared. While the datasets varied in their experimental designs and sample characteristics, cross‐comparison was enabled by applying the Running Fisher algorithm on the BaseSpace platform [43]. This non‐parametric, rank‐based statistical method evaluates similarity between two gene sets by incorporating both the rank order (based on absolute fold change) and the direction of expression changes in DEGs [43]. Greater similarity in gene expression patterns between two datasets is reflected by a lower overlap *p*‐value [44]. Overlap *p*‐values for the RNA‐seq/microarray SD datasets compared with neuronal hyperexcitation datasets are provided in Table S3, and those for the single‐cell SD datasets are shown in Table S4.

### 2.11 Sleep Deprivation Index

The overlap *p*‐values obtained for each comparison between hyperexcitation and SD datasets were transformed into a similarity index using the following formula [20, 63, 64, 65]:

Sleep deprivation index = −log10 (summed overlap *p*‐value between hyperexcitation‐associated genes and SD‐associated genes from the ZT6 SD dataset of the first SD study) × (direction of the overlap). The direction of the overlap is calculated by BaseSpace based on whether the gene expression patterns of the two datasets are concordant or discordant. Therefore, if a neuronal hyperexcitation dataset shares the same DEGs as the SD dataset used in the index, but their direction of expression is opposite, then its sleep deprivation index value will be negative. Note that the sleep deprivation index is referred to as “transcriptomic overlap with SD” on the *y*‐axis of Figure 2A–F.

For each kainate and pilocarpine dataset, indices were calculated and plotted in two‐dimensional space to examine how the extent of overlap between their DEGs and SD‐associated DEGs varied with the time elapsed between SI and tissue collection (Figure 2).

### 2.12 Pathway Enrichment Analysis

Pathways and biological groups enriched among the genes of interest were identified using a combination of rank‐based enrichment statistics and biomedical ontology information available on BaseSpace [64, 65, 66]. For the meta‐analysis of bulk transcriptomic data, genes positively correlated with the SD dataset derived from C57/BL6J mice sleep‐deprived for 6 h at ZT6 were extracted from all hyperexcitation datasets, except for latent kainate and pilocarpine datasets (samples collected 24 h or later post‐SI). This procedure yielded a total of 20 “positive‐gene datasets”. These datasets were subjected to BaseSpace biogroup analysis to identify pathways and biological groups that were consistently enriched across the 20 positive‐gene datasets (Figure 3).

### 2.13 Cell Type Analysis

The DEGs associated with SD from various cell types obtained from the hypothalamus and cortex of sleep‐deprived mice [59] were compared with the DEGs associated with various models of neuronal hyperexcitation. The three cell types showing the highest similarity were selected for further analysis (Table S4). For each selected cell type, datasets were constructed by isolating genes positively correlated with each neuronal hyperexcitation dataset that showed a significant overlap *p*‐value. For example, microglia exhibited 30 significant positive overlaps, resulting in 30 positive‐gene datasets. Similarly, 20 positive‐gene datasets were generated for neurons and 21 for endothelial cells (Figure 4). Meta‐analysis was then performed separately for each cell type dataset to identify significantly enriched genes across datasets (Figure 4).

## 3 Results

### 3.1 Sleep Deprivation Datasets Show Positive Transcriptomic Overlap for Various Neuronal Hyperexcitation Models

**FIGURE 1:**
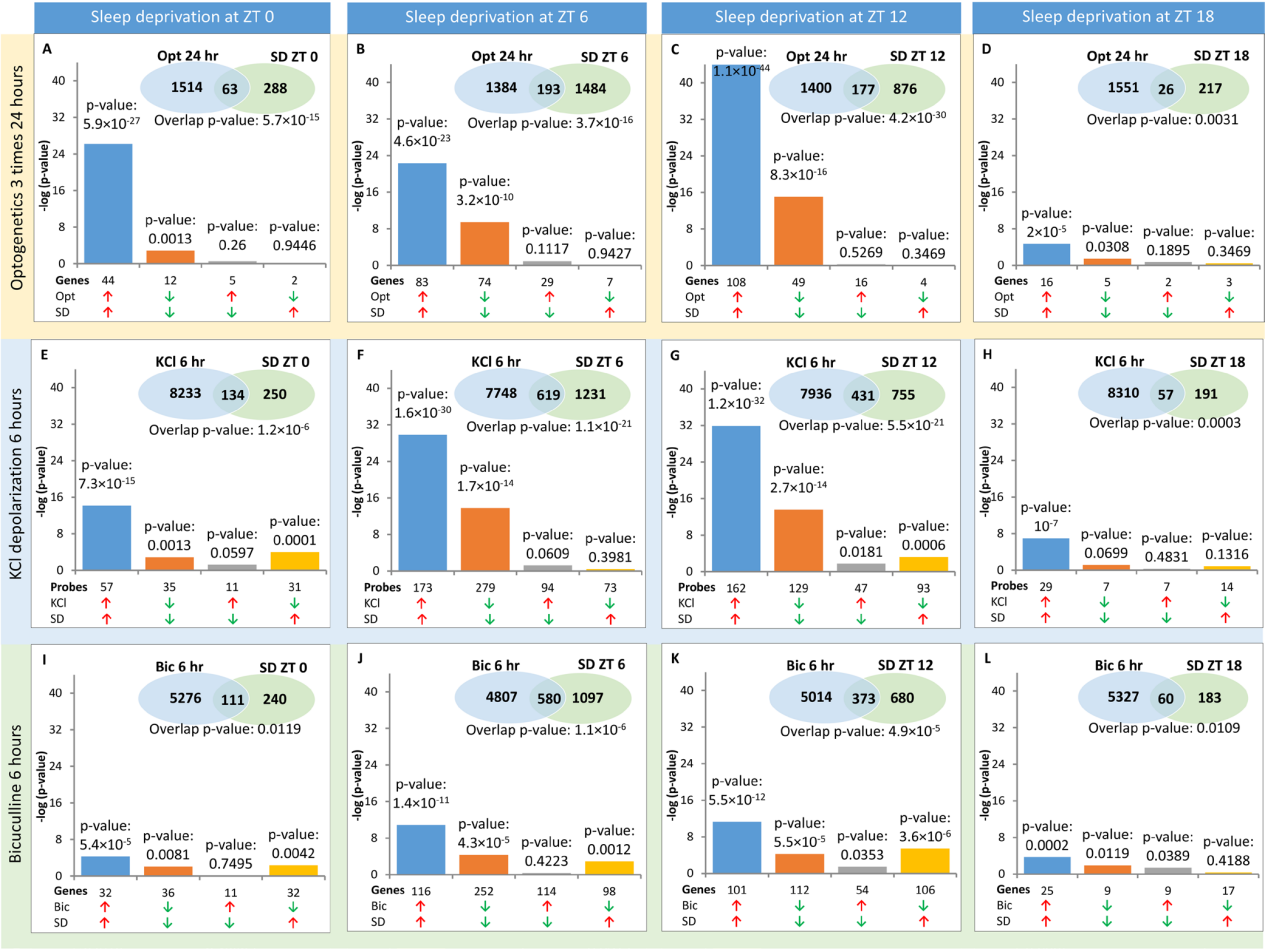
Overlap between SD and different models of neuronal hyperexcitation. On the left of each row, the neuronal hyperexcitation model utilized is indicated. On top of each column, the time at which SD was initiated is indicated. The SD mice lived in a 12‐h light and 12‐h dark enclosure, with ZT0 corresponding to the start of the light phase. Within each subfigure, the title of each neuronal hyperexcitation model was abbreviated: Opt for optogenetically‐induced hyperexcitation, KCl for KCl depolarization‐induced hyperexcitation, and Bic for bicuculline‐induced hyperexcitation. The four SD datasets utilizing C57 mice from the first SD study were used for the comparisons shown in the figure. The Venn diagrams indicate the comparison of hyperexcitation‐associated genes/probes with SD‐associated genes/probes. The Bar graphs illustrate the *p*‐values for the overlap of genes/probes regulated in the same direction (upregulated: blue bars; downregulated: orange bars) under both conditions or in opposite directions (gray and yellow bars) in each condition. Genes/probes with red arrows are upregulated in their respective datasets, whereas those with green arrows are downregulated. Note that the KCl depolarization transcriptomic data (E–H) was produced using microarrays and therefore the panels display probe overlaps rather than gene overlaps with the SD datasets.

Transcriptomic analysis revealed a significant overlap in gene expression changes between SD and multiple neuronal hyperexcitation models, with stronger positive overlap when SD was initiated at ZT0 to ZT12 rather than ZT18 (ZT0 to ZT12: light phase; ZT18: dark phase) (Figure 1). Here and throughout this study, “positive transcriptomic overlap” refers to statistically significant same‐direction differential gene expression overlap as assessed by the Running Fisher algorithm, rather than correlation of continuous expression values. Each SD dataset was compared with various neuronal hyperexcitation models using the Running Fisher algorithm. Figure 1 summarizes these overlap analyses across optogenetic, KCl depolarization, and bicuculline‐induced neuronal hyperexcitation models [48, 51, 52, 56, 57, 58].

Out of 1577 genes altered in the dataset of optogenetically stimulated mice (DEGs from mice 24 h after being stimulated 3 times compared to unstimulated mice), 63 were shared with ZT0‐initiated SD‐associated genes (DEGs in mice sleep‐deprived at ZT0 compared to non‐sleep‐deprived mice). The overlap *p*‐value between the two conditions was 5.7 × 10^−15^, indicating significant positive overlap in gene expression changes (Figure 1A). Among the 63 shared genes, 44 were upregulated (*p* = 5.9 × 10^−27^) and 12 were downregulated (*p* = 0.0013) in both conditions (Figure 1A). These genes were classified as directionally concordant and contributed to the observed positive transcriptomic overlap. Furthermore, 5 genes were upregulated in optogenetically stimulated mice and downregulated in sleep‐deprived mice (*p* = 0.26), and 2 genes were downregulated in optogenetically stimulated mice and upregulated in sleep‐deprived mice (*p* = 0.9446) (Figure 1A). Genes showing opposite directions of change were classified as directionally discordant and did not contribute to positive overlap. Meta‐analyses using the datasets of each column of Figure 1 revealed the number of hyperexcitation genes differentially expressed in the same direction across datasets varied depending on SD initiation (Table S5). ZT 0, 6, 12 and 18 showed 5, 26, 26, and 4 genes which were shared with all 3 hyperexcitation models (Table S5).

Similar analyses were performed for different SD initiation time points and for different neuronal hyperexcitation models, including KCl depolarization and bicuculline (Figure 1B–L). Across all neuronal hyperexcitation models, positive overlap with SD was stronger when SD was initiated at ZT0 to ZT12 (significant for 153 out of 180 comparisons; Table S3), whereas for ZT18 the *p*‐values were higher and often non‐significant (significant for 19 out of 30 comparisons; Table S3). These results indicate that SD initiated during ZT0 to ZT12 shares a greater proportion of same‐direction DEGs with neuronal hyperexcitation datasets than SD initiated at ZT18.

### 3.2 Time‐Dependent Transcriptomic Overlap Between Sleep Deprivation and Neuronal Hyperexcitation Is Linked to Sample Collection Time Post‐Seizure Induction/Post‐Final Stimulation

**FIGURE 2:**
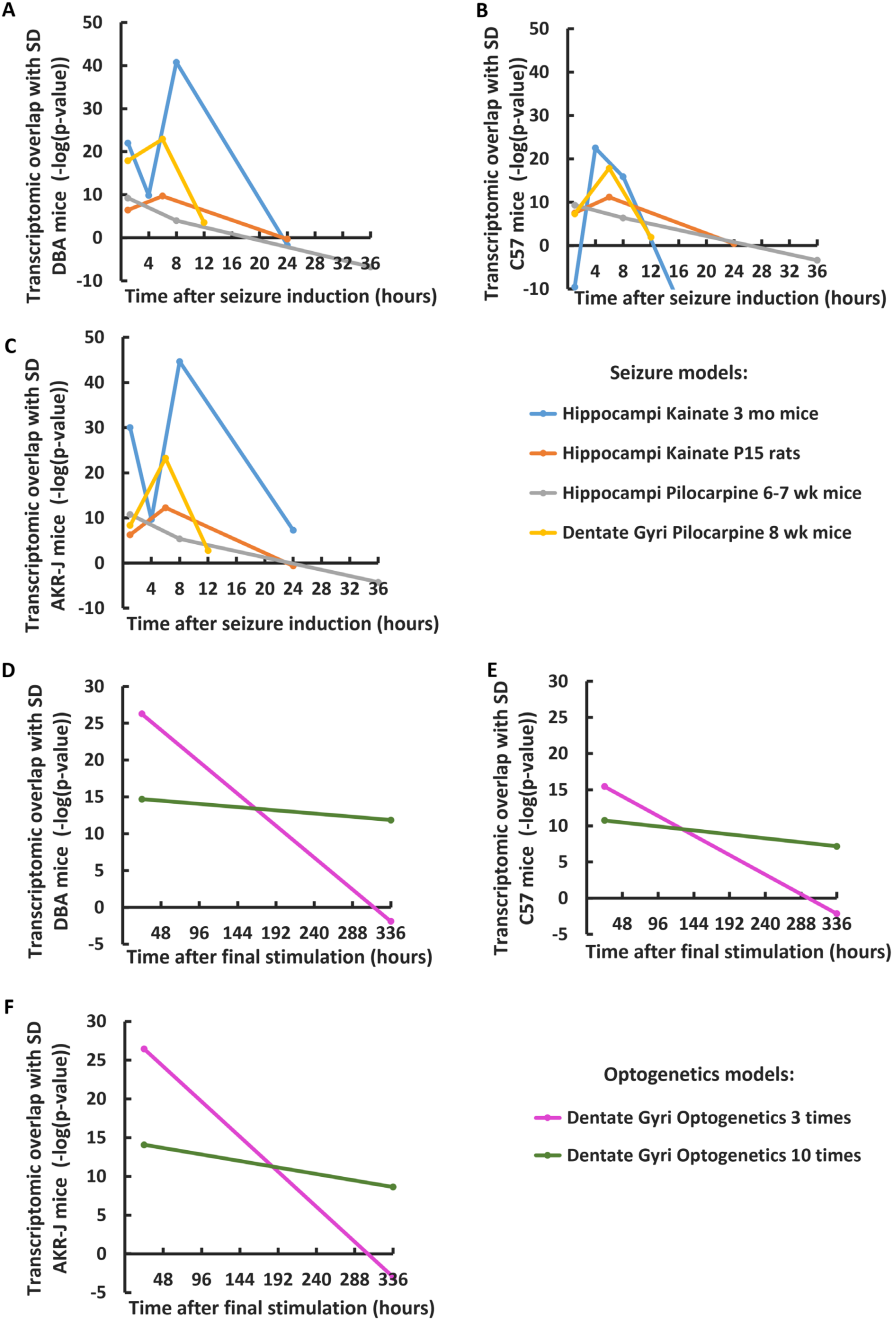
Time post‐seizure induction/stimulation‐dependent overlap between SD and neuronal hyperexcitation models. The figure shows the −log(*p*‐value) for six different models of neuronal hyperexcitation in mice sleep‐deprived for 6 h at ZT6, across different time points post‐seizure induction (SI) or after the final optogenetics stimulation. The hyperexcitation models used are: (1) hippocampi from 3‐month‐old mice treated with kainate (blue line; 1, 4, 8, and 24 h post‐SI), (2) hippocampi from P15 rats treated with kainate (orange line; 1, 6, and 24 h post‐SI), (3) hippocampi from 6 to 7‐week‐old mice treated with pilocarpine (gray line; 1, 8, and 36 h post‐SI), (4) dentate gyri from 8‐week‐old mice treated with pilocarpine (yellow line; 1, 6, and 12 h post‐SI), (5) dentate gyri from 3 to 4‐month‐old mice optogenetically stimulated a total of three times (purple line; 24 and 336 h post final stimulation), and (6) dentate gyri from 3 to 4‐month‐old mice optogenetically stimulated a total of 10 times (green line; 24 and 336 h post final stimulation). The *y*‐axis represents the SD index, whereas the *x*‐axis indicates the hours after SI. Each subfigure corresponds to the log(p‐value) of the hyperexcitation datasets for a different strain of sleep‐deprived mice: DBA (A, D), C57 (B, E), and AKR‐J (C, F). The sign of the −log(*p*‐value) was adjusted according to the direction of the correlation between datasets; positive values indicate that shared DEGs are regulated in the same direction, whereas negative values indicate that shared DEGs are regulated in opposite directions. The SD datasets are from the first SD study.

Since different seizure phases exhibit distinct gene expression changes, seizure phases showing greater transcriptomic similarity to SD were identified using time‐course data following seizure induction (SI) [40, 67, 68]. Comparison of SD with kainate, pilocarpine, and optogenetic neuronal hyperexcitation models showed stronger positive transcriptomic overlap at early time points after SI or stimulation, with reduced overlap at later time points (Figure 2) [40, 53, 54, 55]. The top overlapping genes identified at each time point post SI/stimulation are shown in Table S6.

For example, 3‐month‐old mice treated with kainate displayed significantly positive overlap for SD initiated at ZT6 when samples were collected within 8 h after SI (Figure 2A–C), whereas when samples were collected 24 h after SI, the overlap was predominantly non‐significant or negative (Figure 2A–C). Notably, at 8 h post‐SI these mice showed significant positive overlap for all SD datasets (21 out of 21 datasets; Table S3), while the 24‐h post‐SI dataset was significantly negatively correlated for 13 out of 21 SD datasets and significantly positively correlated for only 3 datasets (Table S3). Similarly, a kainate model using juvenile rats and the pilocarpine‐induced models of hyperexcitation showed significant positive overlap with SD when samples were collected within 12 h post‐SI, and non‐significant or significant negative overlap for samples collected 24 h post‐SI (Figure 2A–2C). The juvenile rat model further served as a sensitivity test, showing that the same time‐dependent correlation with SD persists across different species and developmental stages.

An exception to this trend was observed in optogenetically induced hyperexcitation models, which showed significant positive overlap with SD even 2 weeks post‐stimulation depending on the protocol (Figure 2D–F). The optogenetics datasets utilized mice treated 3 or 10 times in total, with each treatment corresponding to 1 day. When mice had been stimulated 3 times, they showed significant positive overlap with SD 24 h after the final stimulation but negative overlap after 2 weeks. In contrast, mice receiving 10 stimulations showed significant positive overlap even after 2 weeks, suggesting that repetitive stimulation results in longer‐lasting transcriptomic changes that resemble, at the level of differential gene expression, those observed following SD (Figure 2D–F). These findings indicate that SD‐associated DEG overlap is strongest with early post‐seizure or post‐stimulation transcriptional states, whereas repeated stimulation protocols are associated with more persistent same‐direction DEG overlap.

### 3.3 Properties of Neuronal Hyperexcitation Genes Expressed in Sleep‐Deprived Mice

**FIGURE 3:**
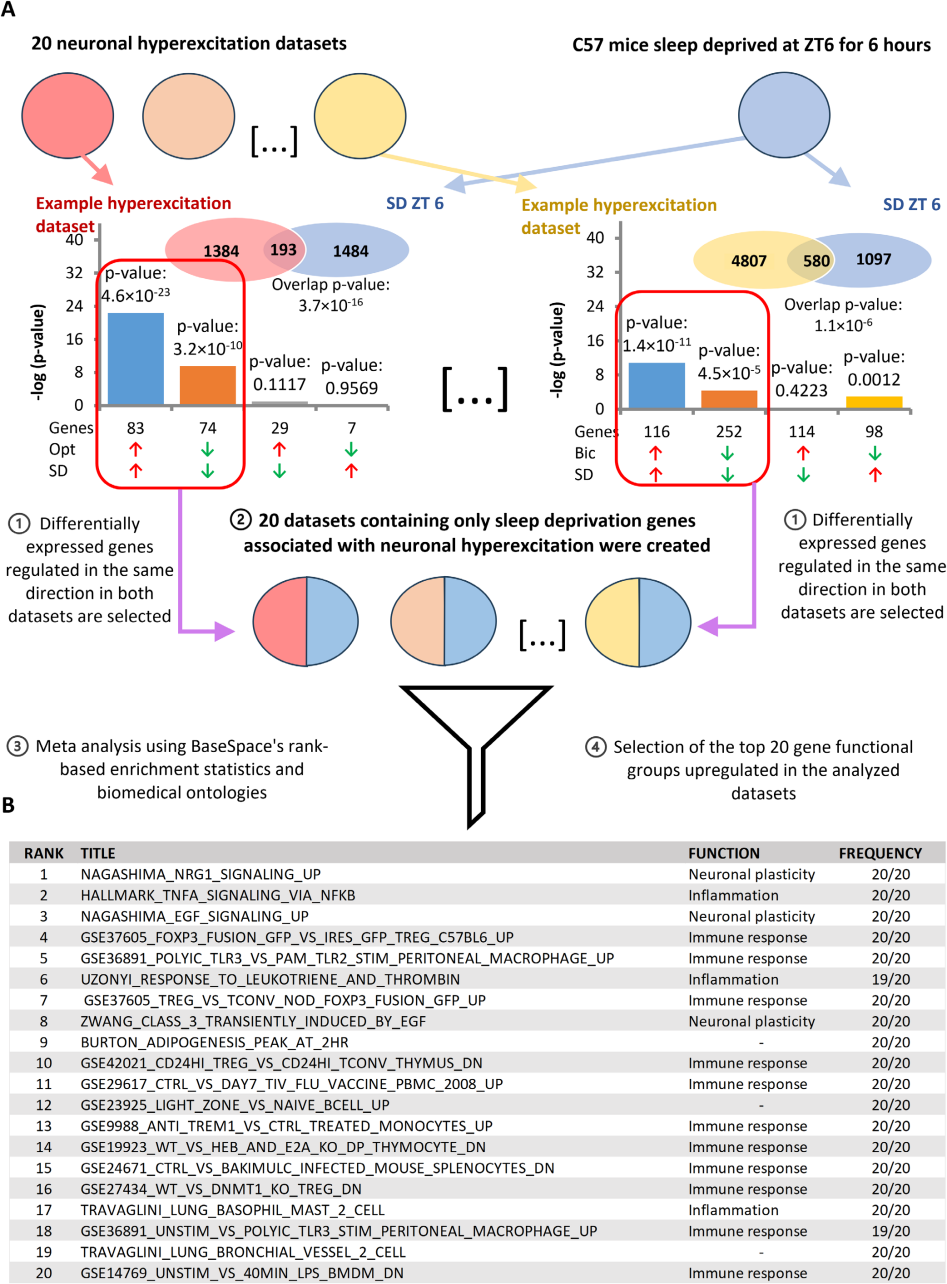
Shared gene functional groups between neuronal hyperexcitation and SD DEGs. (A) Schematic illustration of the meta‐pathway analysis: All neuronal hyperexcitation datasets, with the exception of latent kainate and pilocarpine datasets, were compared with C57 mice sleep‐deprived at ZT6. New datasets were created by extracting only the genes that were (1) common between the two datasets and (2) shared the same direction of expression. The result was 20 datasets containing neuronal hyperexcitation genes positively correlated with SD. These datasets were analyzed on BaseSpace to identify the gene functional groups most closely associated with these datasets. (B) Results of the meta–pathway analysis: The top 20 terms based on the meta‐analysis scores are shown, with the first column indicating their rank in terms of overlap, the second showing their title in BaseSpace, the third providing a brief description of their function, and the fourth showing the number of datasets with which the gene functional group is significantly correlated. All functional groups shown are upregulated in the datasets.

To characterize the transcriptomic pathways associated with genes shared between SD and neuronal hyperexcitation (directionally concordant DEGs), pathway enrichment analysis was performed in BaseSpace to rank gene functional groups shared by the two conditions [40, 48, 51, 52, 53, 54, 55, 56, 57, 58] (Figure 3A). Among the top 20 functional groups, 10 were associated with immune response, three with neuronal plasticity, and three with inflammation (Figure 3B).

Within the top 20 most highly ranked overlapping genes, immediate early genes (IEGs) associated with plasticity, stress response, and inflammation were the most dominant functional group. IEGs such as Junb, Fos, Egr1/2, and Bdnf were upregulated in most datasets included in the analysis (Table 1) [69, 70, 71, 72, 73]. Several upregulated genes, including Ctgf and Ptgs2, have previously been associated with neuronal injury, with reports describing their expression in glial and endothelial cells under injury‐related conditions [74, 75]. In addition, several genes previously associated with neuronal immaturity or stress‐related transcriptional states were present among the overlapping DEGs. In particular, Gadd45b/g, Dusp1, Ctgf, and Tiparp are genes whose upregulation is associated with neuronal immaturity, whereas upregulation of Cdkn1a is associated with cellular senescence (Table 1) [76, 77, 78, 79, 80, 81]. These results suggest that the transcriptomic overlap observed between SD and hyperexcitation‐associated datasets is characterized by enrichment of immune response, neuronal plasticity, and inflammation‐related functional categories, along with prominent IEG involvement.

**TABLE 1:** Shared DEGs between neuronal hyperexcitation and SD.

Rank	Gene name	Significance
1	Junb	17/20
2	Fos	16/20
3	Cdkn1a	16/20
4	Egr4	16/20
5	Gadd45b	14/20
6	Ptgs2	14/20
7	Ctgf	14/20
8	Bdnf	14/20
9	Homer1	14/20
10	Dusp1	13/20
11	Egr1	13/20
12	Klf4	13/10
13	Gadd45g	13/20
14	Crem	13/20
15	Nr4a1	13/20
16	Tiparp	13/20
17	Dusp5	13/20
18	Nr4a2	12/20
19	Egr2	12/20
20	Arc	12/20

*Note:* Twenty datasets containing neuronal hyperexcitation genes associated with SD were analyzed, and the top 20 genes present in the highest number of datasets are shown in the table. The table shows the rank and names of the genes, along with their associations in the context of neurons. The third column includes the number of datasets in which each gene was present.

### 3.4 Cell Types Contributing to the Link Between Sleep Deprivation and Neuronal Hyperexcitation and Their Associated Genes

**FIGURE 4:**
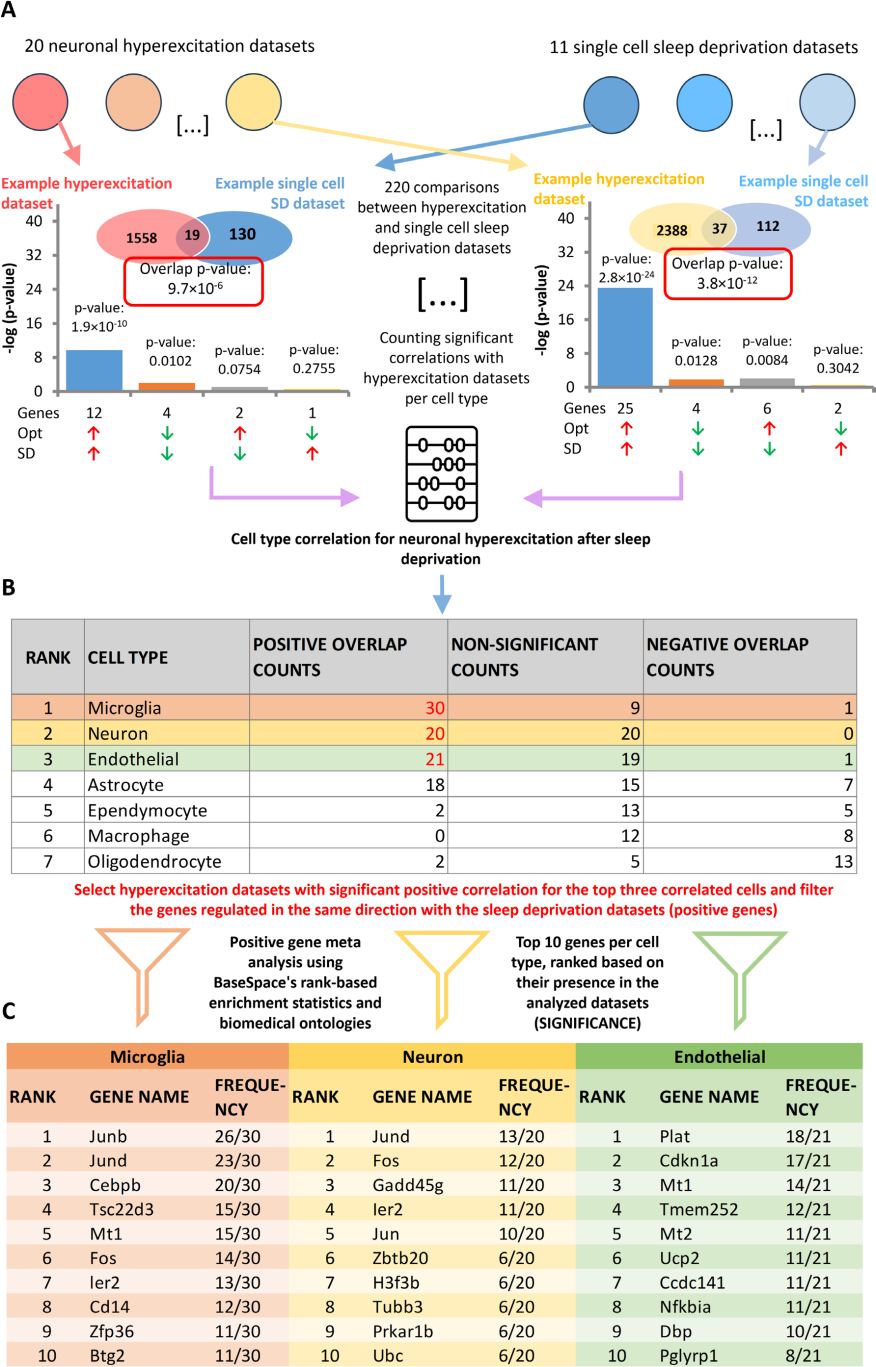
Key cell types contributing to the link between SD and neuronal hyperexcitation. (A) Schematic illustration of the meta‐pathway analysis: Neuronal hyperexcitation datasets were compared with single‐cell SD datasets from the hypothalamus and cortex. (B) Table displaying the number of significant positive, non‐significant, and significant negative overlaps with hyperexcitation datasets for each cell type. When a hyperexcitation dataset was found to have a significant positive overlap with one of these cell types, a new dataset was created by extracting genes from the former that were shared with the latter and that showed the same direction of expression. In total, 30 datasets were created for microglia, 20 for neurons, and 21 for endothelial cells. The newly produced datasets were analyzed by cell type to identify the top 10 significantly expressed hyperexcitation‐associated genes for each cell type. (C) Results of the gene meta‐analysis: The orange portion of the table corresponds to microglia, the yellow portion to neurons, and the green portion to endothelial cells. The first column displays the rank in terms of overlap for the corresponding gene, the second column contains the gene name, and the third column indicates the number of datasets, among those analyzed, in which the gene was significantly upregulated.

To identify the cell types contributing to transcriptomic similarity between SD and neuronal hyperexcitation, scRNA‐seq data from sleep‐deprived mice were analyzed. Cell‐type‐specific differential gene expression profiles were compared with bulk neuronal hyperexcitation datasets using BaseSpace, which enables cross‐platform differential expression analysis and direction‐aware overlap testing across heterogeneous transcriptomic datasets. This approach was used to identify relative enrichment of hyperexcitation‐associated DEGs within SD‐responsive cell types, rather than to assign exclusive cellular origins to bulk expression changes. Single‐cell data from ependymocytes, endothelial cells, neurons, macrophages, oligodendrocytes, microglia, and astrocytes from the hypothalamus and cortex were included [59] (Table S4). While the single‐cell SD data originated from different brain regions (hypothalamus/cortex vs. hippocampus), this cross‐regional comparison allowed for the identification of region‐independent signatures shared between the two conditions. Cell types were ranked based on the number of neuronal hyperexcitation datasets showing significant positive overlap (same‐direction DEG overlap) relative to those showing negative overlap (Figure 4B). The ranking was as follows: microglia, neurons and endothelial cells, astrocytes, ependymocytes, macrophages, and oligodendrocytes (Figure 4B). The top 10 hyperexcitation‐associated genes for each cell type are shown in Figure 4C.

In microglia, these DEGs included IEGs associated with stress response (Jund, Fos, Btg2), immune‐related genes (Junb, Btg2), and inflammation‐associated genes (Cebpb, Zfp36) [69, 70, 71, 82, 83, 84, 85] (Figure 4C). In neurons, IEGs (Jund, Fos, Ier2, Jun) and plasticity‐related genes (Jund, Fos, Jun, H3f3b, Tubb3, Zbtb20) were most prevalent [69, 71, 82, 86, 87, 88] (Figure 4C). In endothelial cells, metabolism‐related genes (Plat, Cdkn1a, Tmem252, Ucp2, Dbp) were the most prevalent group of DEGs [76, 89, 90, 91, 92, 93] (Figure 4C). Together, these results suggest that multiple cell types contribute to SD–hyperexcitation transcriptomic overlap, with distinct functional gene categories enriched across cell types.

### 3.5 Acute Sleep Deprivation Does Not Cause Neuronal Dematuration

Comparison of SD and hyperexcitation datasets with developmental datasets revealed that acute SD alone does not show similarity to transcriptomic immaturity, whereas strong or repetitive hyperexcitation induces a transcriptomic state resembling early development. To determine whether acute SD is sufficient to trigger transcriptomic dematuration (reversion toward immature‐like gene expression), a dataset from 3‐month‐old C57BL/6 mice sleep deprived for 6 h was compared with various developmental datasets (Figure SF1A–D). The SD dataset showed significant negative overlap (greater similarity to adult than juvenile expression) for the P8 vs. Adult (33‐week‐old) mice dataset (*p* = 5.5 × 10^−5^; Figure SF1A), indicating stronger similarity to adult rather than early postnatal mice. In contrast, both the kainate (3‐month‐old mice; Figure SF1E) and optogenetic (3–4‐month‐old mice; Figure SF1I) datasets showed significant positive overlap with early developmental gene expression patterns (*p* = 1.4 × 10^−30^ and *p* = 2.5 × 10^−33^, respectively; Figure SF1E,I). This analysis indicates that acute SD does not produce large‐scale transcriptomic dematuration comparable to that observed following strong or repetitive neuronal hyperexcitation. However, this approach does not exclude the possibility of more subtle, transient, region‐specific, or cell‐type‐restricted transcriptional changes induced by SD.

## 4 Discussion

This study identified that transcriptomic profiles in the brains of sleep‐deprived mice show overlap with transcriptomic signatures reported in multiple mouse models of neuronal hyperexcitation. The transcriptomic overlap between SD and the neuronal hyperexcitation models varied depending on the time of day when SD was performed and the duration after SI. Hyperexcitation models showed the weakest positive overlap for SD when it was initiated at the middle of the mice's active phase (ZT18). Datasets from studies examining the time course of neuronal hyperexcitation indicated that time post‐SI is critical, with SD showing the strongest positive overlap in samples collected 1–12 h post‐SI that largely became non‐significant by ≥ 24 h post‐SI. Pathway analysis of genes contributing to the observed transcriptomic overlaps suggested enrichment of neuronal inflammation, immune response, and neuronal plasticity pathways. Consistent with these findings, the cell‐type analysis indicated that microglia exhibited the strongest contribution to the observed transcriptomic overlaps with neuronal hyperexcitation datasets, followed by neurons and endothelial cells. Genes related to stress and immune responses in microglia, plasticity‐associated IEGs in neurons, and metabolism‐related genes in endothelial cells contributed prominently to the observed overlaps.

### 4.1 Transcriptomic Overlap Between Sleep Deprivation and Neuronal Hyperexcitation Is Tied to the Expression of Immediate Early Genes

The time post‐SI was one of the strongest factors associated with transcriptomic similarity between SD and neuronal hyperexcitation datasets. SD showed predominant positive transcriptomic overlaps with the pilocarpine and kainate datasets whose samples were collected up to 12 h post‐SI. By contrast, datasets collected 24–36 h post‐SI showed largely non‐significant overlaps with SD. The expression patterns of IEGs are a potential candidate explanation for the observed data. Prior reports indicate that the expression of various IEGs such as Bdnf, Arc, and Homer1 peaks within 1–8 h post‐SI and returns to normal levels by the 24‐h mark [40, 94, 95, 96, 97, 98]. The time‐course data for the expression of the aforementioned IEGs align well with the observed time‐course data for the pilocarpine and kainate models. Thus, IEGs likely contribute substantially to the observed transcriptomic similarity between the two conditions, as reflected by their aligned temporal dynamics. The RNA‐seq/microarray gene meta‐analysis showed that IEGs are among the most highly ranked genes shared by the two conditions (Table 1). In this study, SD samples were collected immediately after SD. Therefore, the observed transcriptomic similarity to neuronal hyperexcitation datasets collected at early post‐SI time points may reflect temporal alignment of sampling rather than represent the full temporal dynamics of SD‐induced transcriptomic changes. To test this possibility, future studies will need to include experiments that collect samples at multiple time points following SD.

Repeated optogenetic stimulation was associated with transcriptomic overlap with SD that persisted for up to 2 weeks following the final stimulation (Figure 2D–F). The optogenetic stimulations were modeled after electroconvulsive therapy (ECT), with one stimulation session delivered per day [48]. Mice that were stimulated 3 or 10 times showed significant positive overlap with SD at 24 h after the final stimulation. Moreover, mice stimulated 10 times, but not those stimulated 3 times, continued to show significant positive overlaps even 2 weeks after the final stimulation (Figure 2D–F). Therefore, repetitive stimulation is associated with more sustained transcriptomic changes, with a higher number of repetitions corresponding to longer‐lasting transcriptomic overlaps [48]. IEG persistence may reflect sustained transcriptional signatures driven by the repetitive nature of the stimulation in the optogenetic datasets, in contrast to the single‐induction protocols utilized in the pilocarpine and kainate datasets analyzed in this study. These findings suggest that repetitive neuronal stimulation induces sustained IEG expression, thereby maintaining a transcriptomic profile similar to that of acute SD for extended periods following the final stimulation. Extrapolating from these results, it is possible that chronic or repeated SD could similarly induce long term transcriptomic alterations.

### 4.2 Single‐Cell Analyses Reveal Activity‐ and Metabolism‐Associated Transcriptional Responses

While bulk tissue meta‐analysis established a global transcriptomic similarity between SD and neuronal hyperexcitation, the scRNA‐seq meta‐analysis (Figure 4) provides critical resolution on the cellular drivers of this overlap. The shared transcriptomic signature is not uniformly distributed but is coordinated by distinct cell types, with microglia and endothelial cells also showing prominent overlap with hyperexcitation models. Microglia, which exhibited the highest degree of transcriptomic overlap, upregulated genes associated with immune response and phagocytosis, such as Cd14 and Fos (Figure 4C) [99, 100, 101, 102]. Recent evidence suggests that microglia actively monitor neuronal status and can downscale synapses to dampen excitability [103, 104]. The strong enrichment of hyperexcitation‐associated genes in microglia likely reflects an active response to the SD‐induced “noisy” brain state, potentially attempting to restore homeostasis through neuro‐immune coupling [105, 106, 107].

At the molecular level, sleep deprivation engages metabolic regulatory programs that are not readily apparent from system‐level measurements. Although prior studies utilizing PET imaging or systemic markers report that SD suppresses global glucose metabolism [108, 109, 110, 111], the present analysis revealed a robust upregulation of metabolism‐ and transport‐related genes (e.g., Plat, Ucp2, Dbp) specifically in endothelial cells (Figure 4C) [89, 92, 112]. This discrepancy implies a compensatory neurovascular response: while system‐level metabolism is inhibited, the neurovascular unit may be locally hyperactive to meet the acute energy demands of hyperexcited neurons and to increase blood supply. Notably, Ucp2 was consistently upregulated in endothelial cells; given its role in mitigating reactive oxygen species (ROS), this suggests that the vasculature is not only fueling the hyperexcited brain but also actively defending against the oxidative stress generated by prolonged wakefulness [113].

Together, these single‐cell insights suggest that the “hyperexcitation‐like” state induced by SD is not merely a neuronal phenomenon but a coordinated multicellular adaptive response. Neurons exhibit the transcriptomic signature of hyperactivity (IEGs), while microglia and endothelial cells utilize compensatory pathways to manage the resulting synaptic load and metabolic demand.

### 4.3 Acute Sleep Deprivation Does Not Cause Extensive Neuronal Dematuration

Beyond the immediate transcriptomic similarities described above, we investigated whether SD induces “neuronal dematuration,” a process in which mature neurons revert to an immature‐like molecular state [114, 115]. We have previously proposed the hypothesis that chronic neuronal hyperexcitation acts as a driver for this dematuration, potentially underlying the “immature dentate gyrus” phenotype widely observed in mouse models of various neuropsychiatric disorders [114]. In the present study, SD was found to upregulate several genes associated with neuronal immaturity in both the RNA‐seq/microarray and scRNA‐seq meta‐analyses (Table 1 and Figure 4). These genes include Egr1/4, which are expressed in newborn hippocampal neurons in adults; Klf4, expressed in neural progenitor cells; Gadd45g, expressed widely in the developing cerebellum; and Zbtb20, expressed in immature hippocampal neurons [116, 117, 118, 119, 120]. Nevertheless, the overall transcriptomic profiles triggered by acute SD were not significantly similar to those of younger mice (Figure S1A–D). In contrast, both the kainate and optogenetic models of hyperexcitation showed significant transcriptomic similarity to younger mice (Figure S1E–L), suggesting that the intensity and repetition of stimulation could be critical factors for triggering transcriptomic dematuration. As demonstrated by kindling models, even small imbalances in excitation, when sustained, can significantly impact brain circuits [30, 31]. While this study did not find strong transcriptomic evidence that acute SD induces large‐scale neuronal dematuration, it does not exclude the possibility that chronic or repeated SD could drive such a regression. Investigating whether chronic sleep loss leads to dematuration remains an important future direction for understanding the mechanistic link between sleep disorders and neuropsychiatric pathology.

### 4.4 From Acute Signals to Chronic Circuit Remodeling

While this study focused on acute SD, the relevance of sleep loss to neuropsychiatric disorders lies primarily in chronic conditions. Our optogenetic analysis (discussed in Section 1) provides a conceptual bridge, suggesting that repetition is a catalyst that converts transient molecular responses into sustained alterations [48]. Based on this, it is tempting to hypothesize that chronic SD does not merely prolong the acute state but, through repetitive engagement of specific pathways identified here, drives cumulative circuit remodeling.

The single‐cell meta‐analysis highlights potential drivers of this process. In microglia, the upregulation of Cd14 and Fos (Figure 4) suggests that chronic activation could facilitate sustained phagocytic activity, potentially leading to persistent synaptic modifications [99, 100, 101, 102, 121]. Linked to these synaptic changes, neurons upregulated plasticity‐related IEGs such as Jun and Zbtb20, which are associated with synaptic long‐term potentiation [88, 122]. Furthermore, the accumulation of the histone variant H3f3b (Figure 4C) may indicate an overall increase in baseline transcription in neuronal cells [123].

Extending beyond the neuronal circuitry itself, endothelial cells showed upregulation of genes associated with angiogenesis (Dbp), extracellular matrix remodeling (Plat), and cellular senescence (Cdkn1a) [76, 89, 93]. These changes imply that repeated SD could reconfigure the neurovascular unit, modifying the metabolic support system required for neuronal homeostasis.

However, evidence from acute models indicates that these molecular changes are transient. Following a single event of neuronal excitation or acute SD, expression levels of these genes typically return to baseline, and no lasting structural change may occur. The failure to renormalize could be driven not only by the repetition of these signals but also by the intensity of excitation, alterations in the basal molecular state, and individual differences in resilience.

Collectively, it is tempting to hypothesize that under conditions of chronic or repeated SD, these initially transient responses may accumulate to establish a distinct, remodeled circuit state. Such a state might be characterized by altered information processing, resembling the phenotype observed in the chronic phase of our optogenetic model [48]. This possibility parallels clinical observations in humans, where chronic sleep loss is often associated with changes in cognitive function, such as deficits in spatial memory [124, 125]. To test this hypothesis, future studies must prioritize the acquisition of comprehensive transcriptomic and behavioral data, including detailed time‐course analyses, specifically under conditions of chronic SD.

### 4.5 Acute Sleep Deprivation May Share Therapeutic Mechanisms With Electroconvulsive Therapy

In contrast to the negative consequences of chronic sleep loss discussed above, acute SD has been explored as a therapeutic intervention. SD has been proposed as a potential rapid‐acting treatment for several neuropsychiatric disorders, including depression and post‐traumatic stress disorder, although its clinical efficacy remains controversial and not yet fully established [126, 127]. ECT is among the most effective treatments for severe depression [128, 129]. Our meta‐analysis revealed that SD transcriptomes showed significant positive overlap with the optogenetic datasets derived from the 10‐times stimulation protocol [48]. Since this protocol was designed to model ECT, these results raise the possibility that SD and ECT may engage overlapping transcriptomic programs, potentially through the induction of shared hyperexcitation‐associated pathways.

A major limitation of current SD therapies, which typically rely on a single night of deprivation, is the transient nature of their antidepressant effects; clinical improvements often diminish within 2 weeks [126, 130, 131]. This decay mirrors the transient transcriptomic changes observed in our acute hyperexcitation models. Our finding that repetitive stimulation (i.e., the ECT model) induces sustained transcriptomic alterations suggests that modifying SD protocols, such as employing partial but repeated deprivation, might be necessary to prolong therapeutic benefits. However, this strategy carries inherent risks. Unlike controlled therapeutic interventions, chronic SD in animal models consistently induces depressive‐like behaviors [132, 133, 134, 135]. In humans, chronic sleep insufficiency is increasingly recognized as a transdiagnostic risk factor that exacerbates or predicts the onset of various psychiatric conditions, including schizophrenia, bipolar disorder, and anxiety disorders, as well as Alzheimer's disease [136, 137, 138, 139, 140, 141]. Thus, while repeated SD might hold therapeutic potential by mimicking the sustained molecular effects of ECT, determining the precise boundary between therapeutic hyperexcitation and pathological chronic stress remains a critical challenge for future clinical translation.

### 4.6 Study Limitations

There are various limitations to consider when interpreting the results of this study. The most important limitation is the lack of chronic SD datasets. In clinical practice, chronic SD holds greater relevance than acute SD, as the link to neuropsychiatric disorders stems from persistent sleep disturbances rather than isolated instances of sleep loss [3, 9, 142]. Moreover, no analysis was carried out to account for potential confounding factors. These factors include differences between datasets such as sex, age, duration of SD, and sampling time, all of which could skew the results of this study (Table S2). Age in particular is a factor that could greatly affect the results, considering that the immature nervous system has increased excitability [143]. The extent to which these factors influence the observed results remains unaddressed.

## 5 Conclusion

The results of this study demonstrate a strong transcriptomic convergence between sleep deprivation and models of neuronal hyperexcitation. IEGs emerged as the most widely shared gene functional group, suggesting that SD triggers molecular responses analogous to acute neuronal activation. Beyond this overlap, our single‐cell analysis highlighted distinct cellular contributions: microglia displayed signatures associated with phagocytosis and inflammation, while endothelial cells upregulated genes related to metabolic activation. Although the molecular signatures are strikingly similar, this does not definitively confirm that SD involves the same physiological patterns of neuronal hyperexcitation. Nevertheless, these findings provide a coherent molecular framework linking sleep loss to neuronal functional alterations. Furthermore, the robustly shared genes identified in our single‐cell analysis, associated with synaptic remodeling and transcriptomic regulation, provide a potential roadmap for understanding how acute molecular responses might transition into sustained circuit remodeling. Ultimately, these findings may inform the development of new intervention strategies for sleep‐related neuropsychiatric conditions.

## Author Contributions

H.H. and T.M. developed the study concept. All authors collected, analyzed the data, wrote the paper, and approved the final manuscript.

## Funding

This work was supported by JSPS KAKENHI (Grant JP20H00522, JP25K00903) and MEXT Promotion of Distinctive Joint Research Center Program (Grant FY2018‐2020 JPMXP0618217663, FY2021‐2023 JPMXP0621467949).

## Disclosure

Tsuyoshi Miyakawa is the Editor‐in‐Chief of Neuropsychopharmacology Reports and a co‐author of this article. He was excluded from editorial decision‐making related to the acceptance and publication of this article. Editorial decision‐making was handled independently by the Editor‐in‐Chief, Tsuyoshi Miyakawa, to minimize bias.

## Consent

The authors have nothing to report.

## Conflicts of Interest

The authors declare no conflicts of interest.

## Supporting information


**Figure S1:** Comparison of sleep deprivation and neuronal hyperexcitation models with developmental datasets. On the left of each row, it is indicated whether SD, kainate, or optogenetics data are used for the corresponding comparisons. On top of each column are the titles of the developmental datasets used for the corresponding comparisons. The SD data are from 3‐month‐old mice sleep‐deprived at ZT6 for 6 h, the kainate data are from 3‐month‐old mice sampled 4 h after treament with kainate, and the optogenetics data are from 3 to 4‐month‐old mice that received optogenetic stimulation once per day for 3 consecutive days, with dentate gyri sampled 24 h after the final stimulation. Within each subfigure, the title of each dataset was abbreviated as follows: SD for Sleep Deprivation at ZT 6, Kain for Kainate 4 h, Opt for Optogenetics 3 times 24 h, P8 for P8 vs. Adult (33‐week‐old) mice, P1 for P1 vs. P7 mice, P7 for P7 vs. P15 mice, and P15 for P15 vs. P30 mice. Bar graphs illustrate the *p*‐values for the overlap of genes regulated in the same direction (upregulated: blue bars; downregulated: orange bars) under both conditions or in opposite directions (gray and yellow bars) in each condition. Genes with red arrows are upregulated in their respective dataset, whereas those with green arrows are downregulated. Positive overlap for a developmental dataset such as P8 vs. Adult (33‐week‐old) mice indicates higher overlap for the younger mice (postnatal day 8) compared with the older mice (33 weeks old). Likewise, negative overlap for that dataset indicates higher overlap for the older mice compared with the younger mice.

**Table S1:** Details about the datasets used in this study. The table includes information about the name of the study from which each of the datasets used originate from. GEO ID, age and number of samples associated with each dataset are included.

**Table S3:** Transcriptomic overlap between each bulk RNA‐seq/microarray SD dataset and neuronal hyperexcitation dataset. The table displays the overlap *p*‐value for each SD and neuronal hyperexcitation dataset comparison along with whether that correlation is associated with concordant (+) or discordant (−) gene expression patterns.

**Table S4:** Transcriptomic overlap between each single‐cell SD dataset and bulk RNA‐seq/microarray neuronal hyperexcitation dataset. The table displays the overlap *p*‐value for each SD and neuronal hyperexcitation dataset comparison along with whether that correlation is associated with concordant (+) or discordant (−) gene expression patterns.

**Table S5:** Shared SD genes among optogenetics, KCl depolarization and bicuculine models of neuronal hyperexcitation, across different SD initiation time points. The table is separated into five sheets, each corresponding to a different meta‐analysis. These meta‐analyses are based on and use the same datasets as each column of Figure 1. Thus, the first sheet is titled “ZT 0” and it is a meta‐analysis showing the DEGs shared between SD initiated at ZT 0 and an optogenetics, KCl depolarization and bicuculine model of hyperexcitation. The final sheet titled “All” is a meta‐analysis including all four of the SD datasets used in Figure 1 as well as the three hyperexcitation datasets corresponding to each row of the figure. Red indicates that a gene is significantly upregulated in the corresponding dataset whereas green indicates that it is significantly downregulated.

**Table S6:** Shared SD genes among seizure models or optogenetic models of neuronal hyperexcitation, across different time points post seizure induction/stimulation. The table is separated into six sheets, each corresponding to three meta‐analyses. The three meta‐analyses in each sheet are based on the three different strains of mice sleep deprived at ZT 6 from Figure 2. The sheets are split based on the different time points post SI used in Figure 2A–C or post stimulation used in Figure 2D–F. Thus, the first sheet is titled “1 Hour post seizure induction” and it contains three meta‐analyses showing the top 10 most significant DEGs shared between seizure models of hyperexcitation 1 h post SI and one of the three ZT 6 sleep deprived mouse strains (DBA, C57 or AKR‐J). Red indicates that a gene is significantly upregulated in the corresponding dataset whereas green indicates that it is significantly downregulated.

**Table S2:** Datasets used in each figure. The table includes information about the specific datasets that were used to generate each panel of the figures.

## Data Availability

The datasets presented in this study can be found in BaseSpace (https://basespace.illumina.com/) and the Gene Expression Omnibus (https://www.ncbi.nlm.nih.gov/geo/). All the GEO IDs associated with the datasets are provided in Table S1.
