# Supplementary figures and images for: Sleep Deprivation and Neuronal Hyperexcitation Share Transcriptomic Signatures
Source: Neuropsychopharmacol Rep. 2026 Jun 30;46(3):e70150. doi: 10.1002/npr2.70150 (PMC13318531; doi:10.1002/npr2.70150)

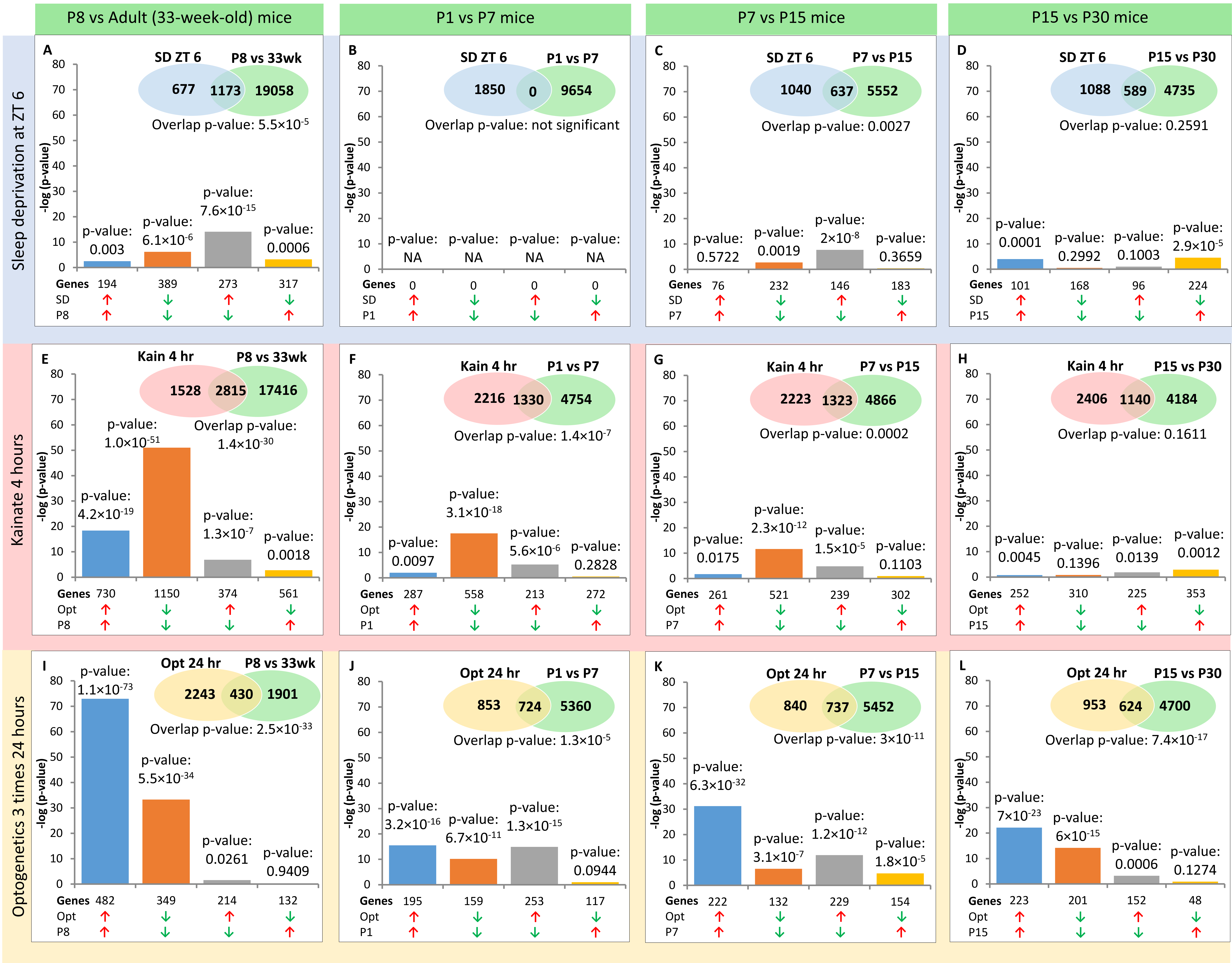

Supplement: Supplementary file 1 — Figure S1: Comparison of sleep deprivation and neuronal hyperexcitation models with developmental datasets. On the left of each row, it is indicated whether SD, kainate, or optogenetics data are used for the corresponding comparisons. On top of each column are the titles of the developmental datasets used for the corresponding comparisons. The SD data are from 3‐month‐old mice sleep‐deprived at ZT6 for 6 h, the kainate data are from 3‐month‐old mice sampled 4 h after treament with kainate, and the optogenetics data are from 3 to 4‐month‐old mice that received optogenetic stimulation once per day for 3 consecutive days, with dentate gyri sampled 24 h after the final stimulation. Within each subfigure, the title of each dataset was abbreviated as follows: SD for Sleep Deprivation at ZT 6, Kain for Kainate 4 h, Opt for Optogenetics 3 times 24 h, P8 for P8 vs. Adult (33‐week‐old) mice, P1 for P1 vs. P7 mice, P7 for P7 vs. P15 mice, and P15 for P15 vs. P30 mice. Bar graphs illustrate the p‐values for the overlap of genes regulated in the same direction (upregulated: blue bars; downregulated: orange bars) under both conditions or in opposite directions (gray and yellow bars) in each condition. Genes with red arrows are upregulated in their respective dataset, whereas those with green arrows are downregulated. Positive overlap for a developmental dataset such as P8 vs. Adult (33‐week‐old) mice indicates higher overlap for the younger mice (postnatal day 8) compared with the older mice (33 weeks old). Likewise, negative overlap for that dataset indicates higher overlap for the older mice compared with the younger mice. [file NPR2-46-e70150-s006.png]
